# In Situ Synthesis of Copper Nanoparticles on Biocarbon Sheets for Surface-Enhanced Raman Scattering

**DOI:** 10.3390/nano15120944

**Published:** 2025-06-18

**Authors:** Jianqiang Wei, Zelong Zhou, Junchao Qian, Yaping Wang, Jun Chen, Yunfei Sun

**Affiliations:** 1 Jiangsu Key Laboratory for Environment Functional Materials, Suzhou University of Science and Technology, Suzhou 215009, China; rolandtheeternity@gmail.com (J.W.); zhouzelong0@gmail.com (Z.Z.); jun666999123@gmail.com (J.C.); 2 Jiangsu Collaborative Innovation Center of Technology and Material of Water Treatment, Suzhou University of Science and Technology, Suzhou 215009, China; 3 UD Keck Center for Advanced Microscopy and Microanalysis, University of Delaware, Newark, DE 19716, USA; wangyp@udel.edu

**Keywords:** surface-enhanced Raman spectroscopy, copper nanoparticle, biotemplate, biocarbon, antibiotics detection

## Abstract

A copper nanoparticles@porous biocarbon substrate was designed for Surface-Enhanced Raman Spectroscopy (SERS) via a simple reduction method. In the detection of three trace antibiotics, the substrate exhibits a very high Raman enhancement efficiency. This is partly because the biocarbon is rich in meso-micropores, which can rapidly trap target molecules. On the other hand, the copper nanoparticles embedded on the surface of the carbon sheets generate a large number of plasmonic hotspots, leading to an increase in Raman signal intensity. These results suggest that this substrate has utility for SERS applications in food safety, medicine, and water pollution detection.

## 1. Introduction

Since its discovery in the 1970s, Surface-Enhanced Raman Spectroscopy (SERS) has been a powerful, rapid detection technique that provides information about the structural properties of a molecule [1,2]. Its main principle is to increase the intensity of the original weak Raman signal by hundreds or even tens of thousands of times when the probed molecules are adsorbed on the rough surface or nanostructure of noble metal substrates (e.g., Au and Ag) [3,4,5]. It enables the highly sensitive structural detection of ultra-low-concentration analytes by amplifying the electromagnetic fields generated by the excitation of localized surface plasmons [6,7].

After years of debate, two distinct mechanisms underlying Surface-Enhanced Raman Scattering (SERS) have gained broad acceptance: the electromagnetic mechanism (EM) and the chemical mechanism (CM). The electromagnetic mechanism arises from the coupling of incident laser light with the localized surface plasmon resonance (LSPR) of nanostructured metal surfaces [8,9]. This interaction generates colossal electromagnetic field enhancements, amplifying the scattering cross-section by up to 10^14^ orders of magnitude. Both theoretical models and experimental studies have provided robust validation for the EM. In contrast, the chemical mechanism involves a charge transfer between the substrate and adsorbed molecules, driven by bonding interactions at the adsorbate–substrate interface. This process enhances the scattering cross-section by approximately 10^2^ orders of magnitude, primarily through changes in the polarizability of the molecule during charge–transfer transitions [10].

Although the phenomenon of SERS has been known for decades, the detection of trace substances such as antibiotics at very low concentrations remains a major challenge. This is because SERS hotspots are often dependent on the nanostructure of the substrate. For example, droplets may slide on the nanostructure surface and make it impossible to effectively concentrate or capture the target molecules to form enough hotspots [11].

Up to now, many studies have focused on porous or reticulated noble metal structures to improve detection sensitivity [12,13]. As a result, the complexity and high cost of preparing gold and silver nanoparticles have made it difficult to extend SERS to a wide range of applications in environmental monitoring, food safety, biotechnology, and other fields [14,15]. In the work presented here, we have developed a SERS substance that has a porous biocarbon structure coated with a fine layer of low-cost copper nanoparticles. This design is easily scaled up using a simple biocarbon reduction method. The obtained SERS substrates showed dramatically improved sensitivity, excellent stability, and reusability in the detection of trace antibiotics. The results of this investigation will provide a new SERS-active nanomaterial for the online detection of organic pollutants and environmental hazards monitoring.

## 2. Materials and Methods

### 2.1. Chemicals

Copper nitrate solution (Cu(NO_3_)_2_·3H_2_O) and ethanol (C_2_H_5_OH, 99%, AR) were purchased from China National Medicines Corporation Ltd. (Shanghai, China). All chemicals were analytically pure and used without further purification in the experimental process. Cherry blossoms were harvested from the campus. Ultrapure water was prepared by purification with a Heal Force system.

### 2.2. Synthesis of Copper Nanoparticles@porous Carbon

Cherry blossom petals were first thoroughly washed several times with ultrapure water. The pretreated petals (about 10 g) were then placed in a mixed solution of ethanol/ultrapure water (*v*/*v*:1/2, 20 °C). In total, 0.241 g of Cu(NO_3_)_2_·3H_2_O was subsequently dissolved in 50 mL of ultrapure water and added to the above solution for 24 h. The petals were then fished out and dried in an oven at 60 °C. Finally, the dry petals were carbonized in a tube furnace at a temperature of 800 °C for 2 h under a nitrogen atmosphere. After the reduction process, the copper substrate was obtained after natural cooling to room temperature.

### 2.3. Characterization Methods

The morphologies of the nanostructures were characterized using Field-Emission Scanning Electron Microscopy (FESEM) images taken on a Sigma 300 microscope (ZEISS, Oberkochen, Germany). Transmission Electron Microscopy (TEM) and High-Resolution Transmission Electron Microscopy (HRTEM) images were obtained using a JEM-2100F microscope (JEOL, Tokyo, Japan) operating at 200 kV. The crystal structure of the Cu nanoparticles@porous carbon sheet was analyzed by an X-ray diffraction system (Bruker D8 Advance, Karlsruhe, Germany). Brunauer–Emmett–Teller (BET) specific surface area and micropore size distribution were measured using an automated adsorption system (ASAP 2460, Atlanta, GA, USA). X-ray photoelectron spectroscopy (XPS) measurement was recorded using a Thermo Scientific (East Grinstead, UK) K-Alpha XPS system with a monochromatic Al Kα source.

### 2.4. SERS Measurements

SERS spectra were acquired on a DXR Raman spectrometer from Thermofischer Scientific with a 532 nm excitation. A 50× objective was utilized to focus the laser beam into a Cu nanoparticles substrate impregnated with an antibiotic solution. Each spectrum was accumulated with 4 s exposures (3 mW) through a 50 μm pinhole.

## 3. Results

**Figure 1:**
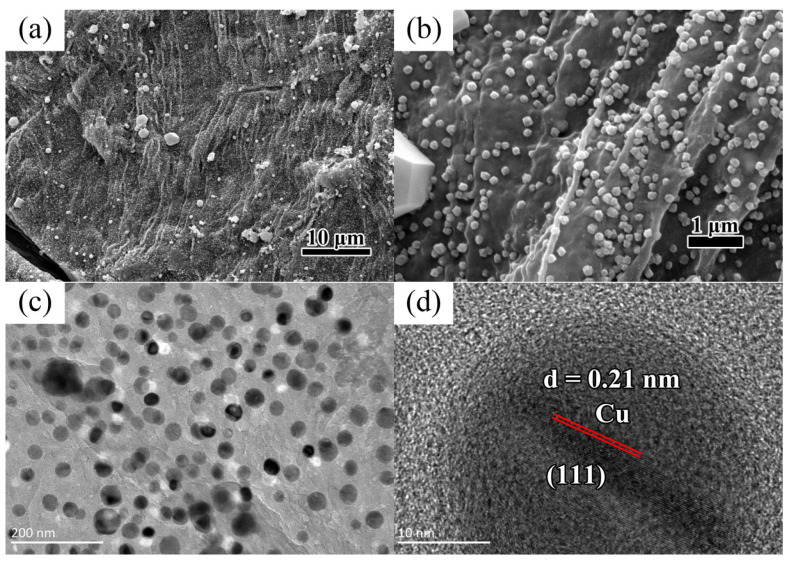
(**a**,**b**) FESEM images of Cu nanoparticles on the biocarbon sheet under different magnifications; (**c**) TEM and (**d**) HRTEM images of Cu nanoparticles.

Figure 1a shows the FESEM image of the Cu nanoparticles on the biocarbon sheet at a low magnification. The large sheet of biocarbon was covered by densely packed copper nanoparticles. Zooming in on Figure 1b, we can see that the copper nanoparticles on the carbon film are uniform in size and evenly distributed. To further identify the particle morphology, we used a transmission electron microscope for observation. As shown in Figure 1c, the nanoparticles are laid flat in a layer of carbon film, and the average size of these nanoparticles is 25 nm. The corresponding HRTEM image (Figure 1d) shows that the clear crystal fringes and the interplanar crystal spacing of about 0.21 nm match well with the (111) planes of Cu (01-070-3038).

**Figure 2:**
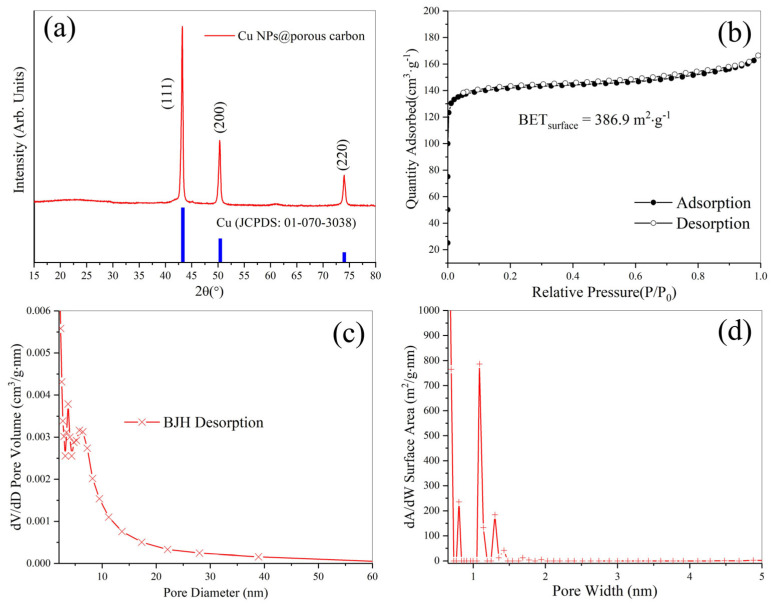
(**a**) X-ray diffraction patterns for copper nanoparticles@porous biocarbon; (**b**) the corresponding N_2_ adsorption–desorption isotherm; (**c**) the relevant BJH desorption pore size distribution; (**d**) micropore size distribution.

X-ray diffraction (XRD) measurements were conducted to analyze the crystal structure of the Cu nanoparticles@porous biocarbon. In Figure 2a, the peaks at 2θ = 43.17, 50.35, and 74.01 are indexed to the (111), (200), and (220) crystal planes of the cubic phase Cu (JCPDS no. 01-070-3038). Based on the Scherrer equation, the crystallite size is about 25 nm, which is consistent with the electron microscopic observations. Figure 2b displays the N_2_ adsorption–desorption isotherm of the sample. It can be seen that there is a peak at the beginning of the adsorption curve, which is a Type I-B isotherm, i.e., carbon materials enriched with a large number of micropores [16]. The BET specific surface area is 386.9 m^2^·g^−1^. The meso-pore size distribution (BJH desorption) curve is shown in Figure 2c. The result indicates that the sample had a mesoporous structure with strong peaks centered at 3.6 nm and 5.7 nm. Since the adsorption potential in micropores is significantly stronger than the van der Waals potential on planar surfaces, the Kelvin equation is not suitable for determining the pore size distribution of the micropores. We adapted the Horvath–Kawazoe method to calculate the micropore size distribution. Figure 2d displays the corresponding micropore size distribution, which is centered approximately at 1.1 nm [17]. This unique meso-micropores structure is extremely favorable for the adsorption of the liquid that needs to be used for enhancement.

**Figure 3:**
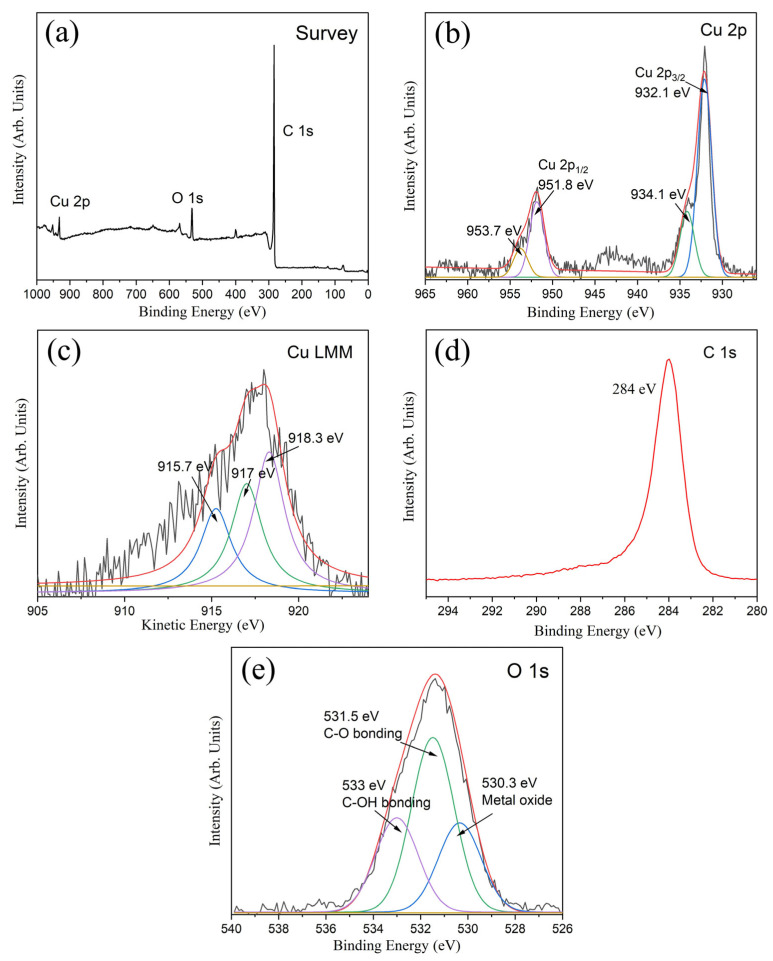
(**a**) XPS survey spectra of copper nanoparticles@porous biocarbon; (**b**) XPS high-resolution spectra of Cu 2p; (**c**) Cu LMM Auger spectrum; (**d**) XPS high-resolution spectra of C 1s and (**e**) O 1s.

The XPS survey spectra of copper nanoparticles@porous biocarbon (Figure 3a) indicate that the samples are mainly composed of Cu, C, and O elements. The spin-orbit splitting of Cu 2p orbitals forms two peaks: Cu(2p_3/2_) and Cu(2p_1/2_). As shown in Figure 3b, the peak corresponding to Cu(2p_3/2_) can be resolved to reach a major peak at 932.1 eV and a minor peak at 934.1 eV. peak. The peak at 932.1 eV could be attributed to the Cu^0^ or Cu(I) state, while the minor peak could be assigned to a Cu(II) oxidation state. To further determine the copper oxidation states, we adopted the Cu LMM Auger spectra, with the corresponding results presented in Figure 3c. Through peak-fitting analysis, the peak at 918.3 eV is assigned to Cu^0^, while the peaks at 917.0 eV and 915.7 eV correspond to the Cu(I) and Cu(II) states, respectively [18]. The aforementioned XPS analysis result, which detects sample composition in micron-scale areas at a depth of 1–3 nanometers, demonstrates a significant presence of metallic Cu^0^ on the sample surface. The C 1s core-level XPS spectrum is shown in Figure 3d, and it is derived from lamellar porous carbon in the samples. Similarly, we performed peak fitting/deconvolution on the O1s spectrum (Figure 3e). The oxygen peak at 530.3 eV corresponds to minor copper oxidation states on the surface, whereas the peaks at 531.5 eV and 533 eV are assigned to the C-O bonds and C-OH (hydroxyl) groups, respectively.

**Figure 4:**
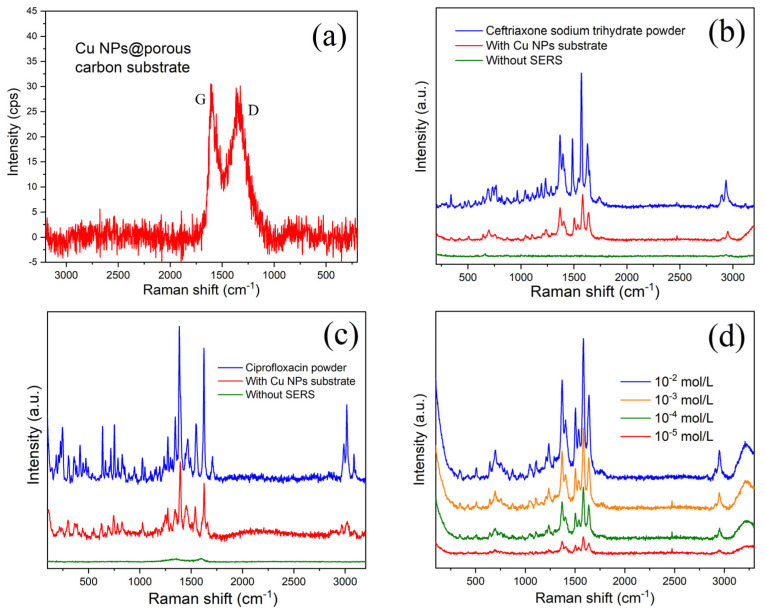

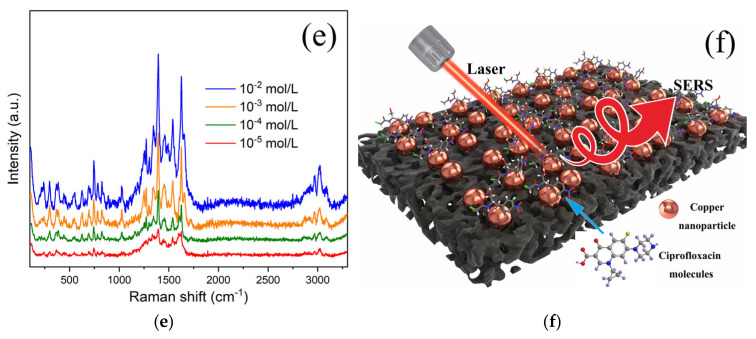
(**a**) Raman spectrum of Cu nanoparticles@porous carbon; (**b**) surface-enhanced Raman spectra of 1 × 10^−3^ mol/L ceftriaxone sodium trihydrate adsorbed on substrate; (**c**) ciprofloxacin adsorbed on substrate; (**d**) SERS responses of ciprofloxacin molecules; (**e**) ceftriaxone sodium trihydrate molecules adsorbed on substrate at different concentrations after soaking for 2 h; (**f**) schematic diagram of the SERS mechanism for copper nanoparticle substrates.

Figure 4a shows the Raman curve of the blank substrate. Two low D and G peaks can be seen, which originate from the breathing modes of six-atom rings and high-frequency E_2g_ phonon from porous carbon. Copper nanoparticles have no signal in this band. This is a good indication that the material we prepared is very suitable as a substrate for SERS. We immersed this porous carbon substrate without doping copper particles in an antibiotic solution of the same concentration for 24 h as the control sample. After subjecting it to Raman detection under identical conditions, we incorporated the results into Figure 4b,c for comparison with the enhanced Raman signals, represented by the green curve. Figure 4b depicts the SER of the spectra of a 1 × 10^−3^ mol/L ceftriaxone sodium trihydrate molecule adsorbed on the substrate. There is no Raman signal for the substrate without Cu nanoparticles (green line) but an obvious Raman signal for Cu nanoparticles@porous carbon, indicating the enhancement effect of copper nanoparticles (red curve). The blue curve in Figure 4b is the Raman signal profile of ceftriaxone sodium trihydrate powder. The peaks are therefore extremely sharp. Compared to the Raman enhanced profile in red, the peak shapes are extremely similar, but the peak positions are slightly shifted, which is also widely experienced in SER reactions. We then tried the SER test with another antibiotic of the same concentration placed on the Cu nanoparticles substrate. Figure 4c shows the SER peaks of ciprofloxacin. It can again be seen that the signals become sharp through the enhancement of the substrate, while both the D and G Raman peaks from carbon can be nearly masked out, leaving the observation unaffected. To further evaluate the potential of Cu nanoparticles@porous carbon membrane as a SERS substrate, we prepared a ceftriaxone sodium trihydrate solution covering the range of 10^−2^ to 10^−5^ mol/L through serial dilution. Figure 4d shows the SERS responses of ceftriaxone sodium trihydrate molecules adsorbed on the substrate at different concentrations after soaking for 2 h. As shown by the red curve in Figure 4d, the Raman peak maximum of the ceftriaxone sodium at 1587 cm⁻^1^ approaches the threshold of being obscured by background signals, resulting in a corresponding limit of detection (LOD) of 10^−7^ mol/L. As shown in Figure 4e, the same phenomenon was observed during the SERS detection of ciprofloxacin. Based on these results, we have developed a schematic diagram illustrating the mechanism of SERS detection of antibiotic molecules using copper nanoparticles (refer to Figure 4f).

Due to the inferior homogeneity of the substrate compared to chemical vapor deposition (CVD)-processed counterparts, the Raman peaks exhibit compromised linear correlation, which is therefore excluded from the current discussion. These experiments fully demonstrate that the obtained substrate can have more significant Raman enhancement effects on some antibiotics. However, the lack of sufficient ordering of the surface structure has led to the current inability to achieve the quantitative detection of antibiotics.

## 4. Conclusions

In summary, we have developed a novel reduction method that combines the porous adsorbed carbon structure with the high Raman enhancement effect of Cu nanoparticles, making Cu nanoparticles@porous carbon highly sensitive SERS substrates. The substrate surface is coated with a layer of copper nanoparticles, with a grain size of 25 nm. The porous carbon structure not only protects the copper nanoparticles but also enhances the adsorption of target molecules. Such a mosaic structure shows high SERS sensitivity to two kinds of antibiotics and is expected to be applied to the trace detection of other types of antibiotics. It can be conveniently and cheaply produced in large quantities, so the unique substrate is expected to expand the applications of SERS to food safety, medicine, and environmental pollution.

## Data Availability

The datasets used and/or analyzed during the current study are available from the corresponding author on reasonable request.
